# Force of Infection Model for Estimating Time to Dengue Virus Seropositivity among Expatriate Populations, Thailand

**DOI:** 10.3201/eid3106.241686

**Published:** 2025-06

**Authors:** Erica Rapheal, Amornphat Kitro, Hisham Imad, Marco Hamins-Peurtolas, Jutarmas Olanwijitwong, Lapakorn Chatapat, Taweewun Hunsawong, Kathryn Anderson, Watcharapong Piyaphanee

**Affiliations:** University of Minnesota School of Public Health, Minneapolis, Minnesota, USA (E. Rapheal); Chiang Mai University, Chiang Mai, Thailand (A. Kitro); Mahidol University, Bangkok, Thailand (H. Imad, J. Olanwijitwong, L. Chatapat, W. Piyaphanee); Osaka University, Osaka, Japan (H. Imad); University of California, San Francisco, California, USA (M. Hamins-Peurolas); US Army Medical Directorate of the Armed Force Research Institute of Medical Sciences, Bangkok (T. Hunsawong); SUNY Upstate Medical University, Syracuse, New York, USA (K. Anderson)

**Keywords:** dengue, viruses, vector-borne infections, travel medicine, vaccines, arboviruses, epidemiological models, Thailand

## Abstract

Dengue is a major cause of illness among local populations and travelers in dengue-endemic areas, particularly those who stay for an extended period. However, little is known about dengue risk among expatriates and other long-term travelers. We used catalytic models of force of infection to estimate time to 60% dengue virus (DENV) seropositivity for a cross-section of expatriates living in Bangkok and Pattaya, Thailand. Our model adjusted for daily time spent outside, years not exposed to DENV, sex, living environment, and use of mosquito repellent, nets, long sleeves, and air conditioning. We estimated an adjusted annual force of infection of 0.014 (95% CI 0.003–0.054) per year spent in dengue-endemic areas (67.3 years to 60% seropositivity), below that of local populations. Our findings suggest that expatriates have a DENV exposure profile distinct from locals and short-term travelers and should likely be considered independently when developing vaccine and prevention recommendations.

Dengue virus (DENV) is a mosquitoborne flavivirus that causes an estimated 100 million infections and >50 million febrile illnesses every year (*1*). Although primary DENV infections are typically clinically mild, secondary infection with a different strain can be severe and can result in thrombocytopenia, plasma leakage, pleural effusion, circulatory failure, and death (*2*–*6*). Half of the global population lives in an area suitable for DENV transmission, and changes in climate and human mobility, and the subsequent expansion of mosquito habitats, will continue to increase the global burden of dengue (*7*–*10*).

The development of new dengue vaccines brings with it the need for updated travel recommendations for protection against DENV infection. Four dengue vaccines have passed phase III trials and 2 have been licensed for use in several countries; many more are in earlier phases of development (*11*–*14*). The World Health Organization currently recommends that countries consider incorporating the Qdenga vaccine (Takeda, https://www.takeda.com) into routine immunization programs for populations with >60% DENV seropositivity by age 9 but does not make specific recommendations for travelers (*15*–*17*). Travelers to DENV-endemic regions have flavivirus exposure histories and associated immunologic profiles distinct from the local populations, which might necessitate unique vaccine and mosquito bite prevention recommendations for travelers, particularly those who plan to stay for extended periods. The number of travel-associated dengue cases has been steadily increasing since 2007, hitting an all-time high in 2019 (*18*).

Long-term expatriates (persons who have moved from other countries but typically do not seek citizenship and might not plan to stay permanently) might be at higher risk for DENV infection than short-term travelers (*19*). Census estimates in 2010 for Thailand counted 80,000 expatriates from the United States, Europe, and Australia living in the capital city of Bangkok alone, representing almost 1% of the city’s total population (*20*). Those persons represent a unique intersection between traditional travelers and native-borne Thai persons because although expatriates (initially) have the naive immunologic profile of a visitor, their daily activities might more closely resemble those of long-term residents (*21*).

An improved understanding of the risks experienced by travelers to dengue-endemic regions is essential to develop safe and effective DENV vaccine usage guidelines for these populations. Although the force of infection (FOI) (the rate at which susceptible persons become infected) among local populations for DENV can be high, risk among travelers might be altered by factors such as increased access to air conditioning and window screens (which might decrease risk), increased immunologic susceptibility to DENV (which might increase risk), or a decreased understanding of local public health threats and mitigation measures for DENV (which might increase risk) (*22*,*23*). Previous studies from this group, including an ongoing cohort study in central Thailand, have indicated that most citizens of Thailand have experienced >1 DENV infection by 6–10 years of age (*23*–*26*).

Previously published findings from a cross-sectional study of expatriates living in Bangkok and Pattaya, Thailand, found DENV seroprevalence to be 11%–21% and showed that expatriates who have been in Thailand for a longer duration are more likely to have DENV-neutralizing antibodies (*27*). In this analysis, we continue that work by contrasting the estimated FOI by years of residence in a dengue-endemic area (DEA) (such as Thailand) among expatriates with that experienced by native-born Thai populations. To estimate the time to first DENV infection, we use catalytic models of FOI using age-stratified DENV seroprevalence data. The purpose of this study is to describe how closely the exposure profile of expatriates resembles that of native residents to target public health messaging and inform optimal-use scenarios for DENV countermeasures in this unique population.

## Methods

### Participant Recruitment

The study population and methods for this study were described previously by Kitro et al. (*27*). In brief, visitors to the Travel Medicine Clinic, Hospital for Tropical Diseases, in Bangkok, Thailand, were actively recruited to participate in this study by a member of the study team staff. Advertisements inviting participants to visit the clinic to enroll in the study were also posted in expatriate groups on social media (i.e., Facebook). Finally, the study team traveled to a meeting of the Pattaya City Expat Club to enroll participants and collect samples on site. Data were collected during December 2017–February 2020. Participants were eligible for inclusion if they were born in a non–flavivirus-endemic region, including the United States/Canada, Europe, the Middle East, Australia/New Zealand, and East Asia (Japan, South Korea, and China); resided in Thailand for >1 year cumulatively over their lifetime; were >18 years of age; had no contraindication to blood draw, no history of blood transfusion in the previous 6 months, and no history of dengue vaccination; and provided informed consent.

### Data Collection

Study data were collected using a short survey, including information about participant demographics, health behaviors, mosquito bite prevention habits and risk factors, history of flavivirus vaccination and infection, and travel history. Approximately 5 mL of blood was drawn by venipuncture from participants who met the inclusion criteria. Laboratory testing was performed at the Armed Forces Research Institute of Medicine in Bangkok. Specimens were tested for neutralizing antibody titers against all 4 DENV serotypes, as well as Japanese encephalitis (JEV) and Zika virus (ZIKV), using plaque-reduction neutralization test (PRNT). The PRNT assay provides a quantitative measure of the antibody response after arbovirus exposure by measuring the inhibition of virus infection. The in vitro study uses a monolayer of C6/36 cells on agar that are infected with virus, leading to cell death and creation of viral plaques. Viruses used in this study were DENV-1 (16007), DENV-2 (16681), DENV-3 (16562), DENV-4 (C0036/06), JEV 0423 (SA-14-14-2, vaccine strain), and ZIKV (SV127/14, Thai isolated strain). When mixed with serum containing antibodies, the infection is inhibited, and plaque number is reduced. Serum dilutions can be compared with a control with no virus-neutralizing antibodies to determine the serum concentration at which a 50% plaque reduction is observed (PRNT_50_) (*28*).

A PRNT_50_ value of >0.5 in any >1 of the 4 DENV serotypes qualified a person as seropositive. Participants were defined as DENV naive if they were negative for all 4 DENV strains, monotypic if they were positive for only 1 DENV strain, and multitypic if they were positive for >2 DENV strains.

### Variables

We calculated time in a DEA by adding cumulative time living in Thailand (in months) and travel of >1 month to dengue-endemic countries. We rounded up time to the nearest year.

We prioritized 6 potential demographic confounders in the analysis for the association between time spent in a DEA and DENV seropositivity: time unexposed (calculated by subtracting time in DEA from age; this covariate was used as a proxy for age to minimize correlation between age and years in DEA), average daily time spent outdoors, sex, marriage to a local of Southeast Asia, employment status, and living environment. Average daily time spent outside was reported in hours. Employment status was categorized into employed or unemployed/retired on the basis of an open-ended survey question. Participants selected urban, suburban, rural, or farm for living environment. Analyses used a binary variable comparing urban to any other response.

We also collected information about how frequently 5 mosquito bite–prevention strategies were used: mosquito repellant, long sleeves, window screens, mosquito netting, and air conditioning. Participants self-reported frequency on a scale of 1 (daily) to 5 (never). We split those measures at the median to create binary variables; we categorized values below the median as more frequent use and values greater than or equal to the median as less frequent use.

### Data Analysis

We estimated FOI by fitting serocatalytic models, using seropositivity to >1 of the 4 DENV serotypes as the outcome. We fit serostatus by using a binomial model with a *cloglog* link function with log(years in DEA) as an offset. We adapted code from Ribeiro dos Santos et al. (*23*) and implemented it using R-INLA, which is syntactically similar to the lm function in R where linear predictors can be incorporated (*29*). We fit a crude model to estimate overall FOI, as well as univariate models incorporating each of the 6 demographic and 5 mosquito prevention variables described and a saturated model incorporating all 11 variables. We also used a 10% backward selection method to develop a reduced model. In this method, variables are removed from the model sequentially. The variable with the least impact on the model is dropped, provided that removing that variable changes the FOI estimate to <10%. We repeated this process until no variables could be removed without >10% change in the FOI. All models included the log(years in DEA) offset term (Appendix). We performed all analyses using R version 4.3.1 (The R Project for Statistical Computing, https://www.r-project.org).

### Sensitivity Analysis

To assess the effect of cross-reactivity between flaviviruses, we performed a sensitivity analysis. We assumed all persons with PRNT_50_ >0.5 for JEV or ZIKV were seronegative for DENV.

### Ethical Considerations

The study was approved by the Ethics Committee of Mahidol University (FTM ECT-019-06), the Walter Reed Army Institute of Research Institutional Review Board, the oversight body for AFRIMS regulatory approval (WRAIR no. 2500), and the University of Minnesota Institutional Review Board, which was the funding source for this study. All study participants provided informed written consent, and all data were anonymized before analysis.

## Results

### Participants

Of 235 participants who met the inclusion criteria, 71 (30%) were seropositive for >1 DENV strain. Of those, 29 (41%) were monotypic and 42 (59%) were multitypic. Ten (4%) participants were JEV positive and 5 (2%) were ZIKV positive (Table 1). Persons who were DENV negative had spent an average of 9 years in a DEA, compared with 14 years for those who were DENV positive (p = 0.02) (Table 2). Approximately 1% (279 of 25,922 months) of the total months in a DEA were attributed to time spent in dengue-endemic countries outside of Southeast Asia.

**Table 1:** Serostatus of participants in study of force of infection model for estimating time to DENV seropositivity among expatriate populations, Thailand*

Serostatus	No. (%) participants		
	Total, n = 235	DENV-negative, n = 164	DENV-positive, n = 71
Dengue			
DENV-negative	164 (70)		
Monotypic†	29 (12)		
Multitypic‡	42 (18)		
Japanese encephalitis			
JEV-negative	225	160 (71.1)	65 (28.9)
JEV-positive	10	4 (40)	6 (60)
Zika			
ZIKV-negative	230	161 (70)	69 (30)
ZIKV-positive	5	3 (60)	2 (40)

*DENG, dengue virus; JEV, Japanese encephalitis virus; ZIKV, Zika virus. †Person was previously infected with only 1 DENV serotype. ‡Person was previously infected with >2 different DENV serotypes.

**Table 2:** Descriptive statistics of 235 expatriate participants in study of force of infection model for estimating time to DENV seropositivity among expatriate populations, Thailand*

Characteristic	Total, n = 235	DENV-negative, n = 164	DENV-positive, n = 71	p value
Median time in DEA (range), y	7 (1–53)	7 (1–37)	10 (1–53)	0.02†
Median age (range), y	66 (23–94)	65 (23–88)	68 (27–94)	0.16†
Median time unexposed (range), y	54 (21–78)	53 (21–78)	54 (23–75)	0.94†
Average time outside daily (range), h	5.0 (0.0–24.0)	6.0 (0.0–24.0)	4.5 (0.0–16.0)	0.21†
Sex				0.75‡
M	186 (84)	129 (84)	57 (83)	
F	36 (16)	24 (16)	12 (17)	
Married to person from Southeast Asia				0.91‡
Yes	64 (27)	45 (27)	19 (27)	
No	171 (73)	119 (73)	52 (73)	
Employment status				0.45‡
Employed	50 (25)	36 (26)	14 (21)	
Unemployed/retired	185 (75)	128 (74)	57 (79)	
Living setting				0.084‡
Urban	197 (84)	133 (81)	64 (90)	
Nonurban	38 (16)	31 (19)	7 (10)	
Mosquito repellent				0.68‡
Frequent	56 (24)	38 (23)	18 (26)	
Infrequent	179 (76)	126 (77)	53 (74)	
Long sleeves				0.2‡
Frequent	112 (48)	83 (51)	29 (41)	
Infrequent	123 (52)	81 (49)	42 (59)	
Window screens				0.8‡
Frequent	94 (40)	65 (40)	29 (41)	
Infrequent	141 (60)	99 (60)	42 (59)	
Mosquito nets				0.83‡
Frequent	72 (31)	50 (30)	22 (32)	
Infrequent	163 (69)	114 (70)	49 (68)	
Air conditioning				0.36‡
Frequent	94 (40)	69 (42)	25 (36)	
Infrequent	141 (60)	95 (58)	46 (64)	

*Values are no. (%) except as indicated. DEA, dengue-endemic area; DENV, dengue virus. †By Wilcoxon rank-sum test. ‡By Pearson χ^2^ test.

Study participants were primarily men (84%) and retired (57%). Participant age ranged from 23 to 94 years; the average age was 61 (median 66) years. Most (60%) participants lived in an urban area, and >1 in 4 (27%) were married to a person from Southeast Asia (Table 2).

### FOI Models

Assuming a static FOI for the entirety of time spent in a DEA and incorporating no other risk factors, we estimated an annual FOI of 0.042 (95% CI 0.033–0.053) (≈23.9 years to first DENV infection). Using this model, 60% seroprevalence is reached after 21.9 years in DEA (Figure). Watanabe-Akaike information criterion (WAIC), a Bayesian measure similar to the Akaike information criterion, was 295.0.

**Figure:**
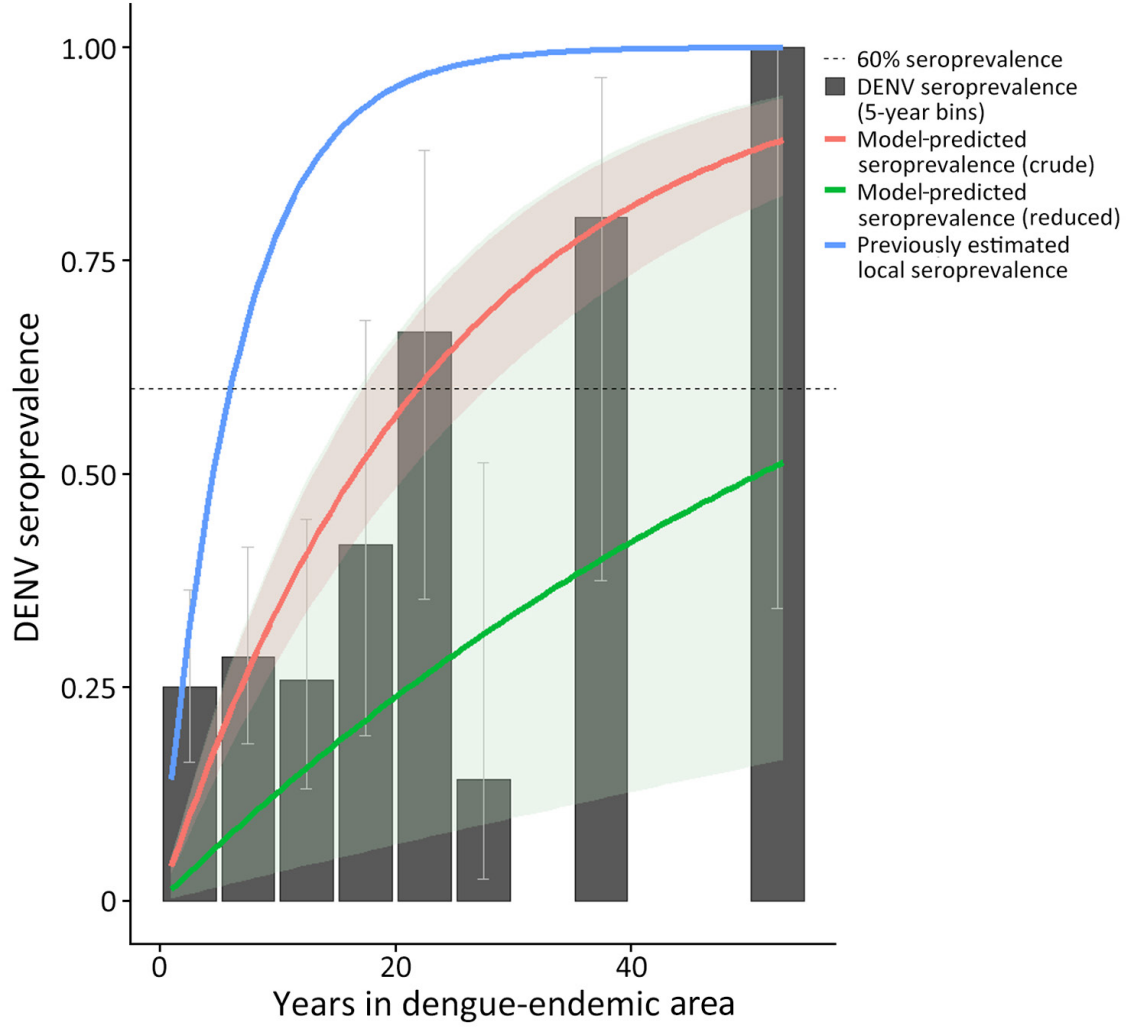
Crude force of infection by years in dengue-endemic area (DEA) in study of force of infection model for estimating time to DENV seropositivity among expatriate populations, Thailand. A serocatalytic model estimating dengue force of infection was fit using a binomial model with a cloglog link function with log(years in DEA) as an offset. Solid red line represents the crude model; solid green line represents the reduced model. The solid blue line represents approximate seroprevalence among locals of Thailand, as modeled by Hamins Puertolas et al. (*25*). Dotted line indicates 60% DENV seroprevalence. Black bars show the seroprevalence of DENV among all study participants in 5-year bins of years spent in a DEA. Uncertainty in measured seroprevalence was calculated using the Wilson confidence interval for proportions (*30*). DENV, dengue virus.

No covariates were significantly associated with DENV seropositivity in univariate models (Table 3). The 10% backward selection method retained years unexposed, male, urban living setting, and frequent use of mosquito repellent, mosquito nets, long sleeves, and air conditioning (Table 4). Only urban living setting was significantly associated with DENV seropositivity in the reduced model (OR 2.45 [95% CI 1.09–5.49]). WAIC for that model was 300.8. A difference of 5.8 between WAIC values provides weak but not conclusive evidence that the crude model is a better predictor than the reduced model (*31*).

**Table 3:** Estimates from univariate logistic models of dengue seropositivity by years in a dengue-endemic area in study of force of infection model for estimating time to dengue virus seropositivity among expatriate populations, Thailand*

Covariate in univariate models	Odds ratio (95% CI)
Years unexposed	1.01 (0.9–1.02)
Average daily time outside, h	0.98 (0.92–1.03)
Male sex	1.24 (0.69–2.25)
Married to native of Southeast Asia	0.75 (0.44–1.22)
Employed	0.72 (0.45–1.48)
Urban living setting	2.04 (0.93–4.49)
Frequent use of mosquito repellent	1.11 (0.65–1.91)
Frequent use of mosquito nets	1.02 (0.61–1.71)
Frequent use of window screens	1.02 (0.63–1.65)
Frequent use of long sleeves	0.66 (0.41–1.07)
Frequent use of air conditioning	0.75 (0.46–1.24)

*Force of infection for years in dengue-endemic area (DEA) is 0.04 (95% CI 0.03–0.06). Serocatalytic models estimating dengue force of infection were fit using a binomial model with a cloglog link function with log(years in DEA) as an offset. Crude model contains only log(years in DEA) offset. Univariate models included 1 covariate and the log(years in DEA) offset.

**Table 4:** Estimates from reduced multivariate model of DENV seropositivity by years in a dengue-endemic area in study of force of infection model for estimating time to seropositivity among expatriate populations, Thailand*

Reduced multivariate model	Odds ratio (95% CI)
Years unexposed	1.01 (0.99–1.02)
Male sex	1.35 (0.74–2.47)
Urban living setting	2.45 (1.09–5.49)
Frequent use of mosquito repellent	1.33 (0.75–2.35)
Frequent use of mosquito nets	1.22 (0.69–2.16)
Frequent use of long sleeves	0.62 (0.36–1.05)
Frequent use of air conditioning	0.77 (0.46–1.30)

*Force of infection (FOI) is 0.01 (95% CI 0.00–0.05). Serocatalytic models estimating dengue force of infection were fit using a binomial model with a cloglog link function with log(years in dengue-endemic area) as an offset. A 10% backward selection method was used to determine which variables were included in the reduced model. Variables were sequentially removed from the model, and the variable with the least impact on the model was dropped. This process was repeated until no variables could be removed without >10% change in the FOI..

Using the reduced multivariate model, we estimated an annual FOI of 0.014 (95% CI 0.003–0.054) (73.5 years to first DENV infection, 67.3 years to 60% DENV seropositivity). Using the reduced multivariate model, a person who is at higher risk for all included variables, where higher risk means associated with higher odds of seropositivity in the reduced model regardless of significance (i.e., lived 60 years in a nonendemic region, male sex, urban living setting, frequent use of mosquito repellent and nets, and infrequent use of long sleeves and air conditioning) would have an estimated annual FOI of 0.101 (95% CI 0.046–0.218). Conversely, a person who is at lower risk for those variables (i.e., lived 17 years in a nonendemic region, female sex) would have an estimated annual FOI of 0.007 (95% CI 0.002–0.025).

### Sensitivity Analysis

Eight persons who were DENV positive in the original analysis were positive for either ZIKV (n = 2) or JEV (n = 6) and were therefore considered DENV negative for the sensitivity analysis. Using those data, we estimated an average annual FOI of 0.037 (95% CI 0.028–0.046) (26 years to 60% seropositive) in the crude model, representing a 17% decrease, and 0.011 (95% CI 0.003–0.049) (82 years to 60% seropositive) in the adjusted model, representing a 21% decrease.

## Discussion

In our study of 235 expatriates living in Bangkok and Pattaya, Thailand, we found a crude DENV FOI of 0.042/year spent in DEA, equating to an estimated 23.9 years to first DENV infection and 21.9 years to 60% DENV seroprevalence. When we adjusted for years unexposed to DENV, sex, living environment, and frequent use of repellent, mosquito nets, long sleeves, and air conditioning, the FOI estimate decreased to 0.014, or 73.5 years to first DENV infection and 67.3 years to 60% DENV seropositivity. Using the adjusted model, a person who is at high risk in all our exposure categories has an estimated FOI per year in DEA of 0.101, >8 times higher than that in the low-risk group (0.007).

Our results suggest that expatriates are a unique population who should be considered as such when developing recommendations for DENV control and prevention. Our average time to 60% seroprevalence was more than twice that for Thai locals, suggesting that expatriates and other long-term travelers might experience a much lower risk for DENV exposure relative to native residents. Data from Rayong Province, which is geographically adjacent to Pattaya, found most persons to be infected by age 10; in other areas, that age is as low as 6 years (*26*,*32*,*33*).

The difference in the estimated annual FOI for high (13.5%) versus low (0.4%) risk participants highlights the need to consider risk when recommending vaccination to the expatriate population. For example, short-term travelers who stay in urban areas might benefit from vaccination during periods of epidemic transmission because of possibly high and transient multitypic cross-protection, but they might experience increased risk with subsequent travel (in the months or early years after vaccination) as immunity wanes. This area bears more study and exploration and was not specifically addressed by Takeda or the manufacturers of the Dengvaxia vaccine (Sanofi Pasteur, https://www.sanofi.com), nor was specific guidance provided by the World Health Organization regarding vaccination of short- or long-term travelers.

The use of mosquito repellents such as DEET and picaridin has been shown to be effective in reducing mosquito bite frequency when applied regularly (*34*,*35*). Those and other primary prevention strategies, as well as community mosquito control measures, have been the recommendations for dengue prevention while awaiting more effective countermeasures for DENV (e.g., vaccines and antiviral drugs) (*2*). Although we did not find a significant association between those prevention measures and DENV seropositivity, the difference in FOI between the groups at low risk and high risk (0.007 for low risk and 0.101 for high risk) suggests that those risk factors might still be drivers of infection risk and underscores the continued importance of nonvaccine interventions. Participants also could have overreported repellant usage to more closely align with recommendations, which would introduce bias into those findings.

Of note, our study population was primarily urban (60%) and male (84%), and the average age was 61 years. This group might be slightly older and have a higher representation of men than the overall expatriate population; a 2021 study from Kitro et al. (*36*) found Western expatriates in Bangkok and Chiang Mai to be 75% men and an average of 56 years of age, and the 2010 census reports that expatriates in Bangkok were ≈64% male (*37*). Bangkok and Pattaya are both attractive retirement spots for Westerners and typically attract an older, more male population (*38*). Therefore, we do consider our study population to be representative of the expatriate population in those communities. Lifestyle differences, including working outside during the day and housing- and infrastructure-related risk factors, were expected to play a role in determining time to DENV infection. However, we found no significant association between DENV infection and age (measured as years unexposed to DENV), employment, or time spent outside in univariate or multivariate analyses (Table 2). We did find an association with urban versus nonurban residence (OR 2.45 [95% CI 1.09–5.49]) in the multivariate analysis. Future studies could examine this relationship in more depth by measuring housing and workplace infrastructure more explicitly.

The first limitation of our study is that our model does not directly take multiple infections into account. PRNT results across DENV serotypes are known to be highly cross-reactive, and it is therefore difficult to assess the number and timing of infections associated with multitypic serostatus (*6*). For this reason, the FOI estimated in this model probably slightly underrepresents the actual value, whereas a model incorporating multiple infections would overestimate the true value. In addition, exposure to cross-reacting arboviruses such as JEV and ZIKV might also affect estimates, something we try to account for, making it difficult to address questions about time to postprimary infection.

Dengue has substantial interannual variation, in both number of cases and case serotype (*39*). Major outbreaks occur regularly in Thailand, most recently in 2015, 2019, and 2023 (*40*). This study does not investigate the possibility of cohort effects related to these outbreak years; future studies might use number of outbreaks experienced as a length-of-stay variable, or weight length of stay by annual disease burden. This study lacked the sample size to power a time-varying FOI analysis, but the use of constant FOI to describe DENV serology is common in hyperendemic regions like Thailand, and the models have been shown to fit DENV data well (*25*,*26*). However, more research is necessary to evaluate whether FOI varies over time, which could help identify periods at which expatriates are at higher or lower risk. Finally, the inherent limitations of a cross-sectional study limit capacity to assess the effects of waning immunity and seroreversion. Previous studies have shown seroreversion rates of <4%, but many unknowns remain about the extent to which primary DENV infections provide lasting immunity (*41*–*43*). In addition, we are not able to determine consistency of covariates over time and are therefore only able to draw conclusions about each person’s current behaviors. A future prospective study should assess how these characteristics, and their associated outcomes, might change over time.

In conclusion, we found that the DENV infection profile of expatriates living in Bangkok and Pattaya, Thailand, does not closely resemble that of native-born residents. The relatively low rate of DENV infection in expatriates suggests that we should consider them to be a group with a distinct exposure profile compared with both local residents and short-term travelers. Future studies examining longitudinal DENV exposure profiles in other native and expatriate populations, and in other regions, will be key to place these findings in a global context. However, our findings should be considered when developing targeted public health messaging and could help inform recommendations for both vaccine and nonvaccine DENV prevention measures.

Appendix Additional information on force of infection model for estimating time to dengue virus seropositivity among expatriate populations, Thailand
