# Supplementary material for: Force of Infection Model for Estimating Time to Dengue Virus Seropositivity among Expatriate Populations, Thailand
Source: Emerg Infect Dis. 2025 Jun;31(6):1149–57. doi: 10.3201/eid3106.241686 (PMC12123937; doi:10.3201/eid3106.241686)
Supplement: Appendix — Additional information on force of infection model for estimating time to dengue virus seropositivity among expatriate populations, Thailand [file 24-1686-Techapp-s1.pdf]

Article DOI: https://doi.org/10.3201/eid3106.241686

EID cannot ensure accessibility for supplementary materials supplied by authors. Readers who have difficulty accessing supplementary content should contact the authors for assistance.

Force of Infection Model for Estimating Time to Dengue Virus Seropositivity among Expatriate Populations, Thailand

Appendix

Equation 1—Catalytic Model

$$p_t = 1 - e^{-\phi t}$$

Where p_t represents the proportion of the population at age t that are seropositive while φ represents the force of infection. Explicitly, we assumed that the probability of testing seropositive at each age was binomially distributed such that $x_t \sim Binomial(N_t, p_t)$, where p_t is the proportion of the population at age t that are seropositive, N_t is the total number of individuals of age t, and x_t is the number of individuals who are seropositive at age t.
